# Public perceptions of people with eating disorders: Commentary on results from the 2022 Australian national survey of mental health-related stigma and discrimination

**DOI:** 10.1186/s40337-023-00786-z

**Published:** 2023-04-16

**Authors:** E. Bryant, S. Touyz, S. Maguire

**Affiliations:** InsideOut Institute for Eating Disorders, Faculty of Medicine and Health, University of Sydney and Sydney Local Health District, Sydney, NSW Australia

**Keywords:** Anorexia nervosa, Bulimia nervosa, Binge eating disorder, Beliefs, Popular opinion, Public view, Disordered eating, Consensus, Sentiment, Opinion

## Abstract

Mental illness is highly prevalent in the community. As such, significant attention has been paid in recent years to raising awareness of the mental health disorders (including eating disorders). This includes efforts to normalise help-seeking, campaigns to reduce stigma and discrimination, targeted research funding and advocacy for improved and accessible mental health service provision. But have these initiatives changed public attitude? The 2022 National Survey of Mental Health-Related Stigma and Discrimination is the first of four national surveys canvassing the general public’s perceptions of people with mental health disorders (including stigmatising and discriminatory beliefs) conducted since 1995 to include eating disorders. It finds significant prejudice against those with mental health disorders still exists within the community, particularly among younger Australians. For eating disorders, this is primarily related to attributions of blame and personal weakness. Findings from the survey are discussed in this commentary.

**Supplementary Information:**

The online version contains supplementary material available at 10.1186/s40337-023-00786-z.

## Introduction

‘The stigmatized individual is asked to act so as to imply neither that his burden is heavy nor that bearing it has made him different from us; at the same time he must keep himself at that remove from us which assures our painlessly being able to confirm this belief about him.’—Erving Goffman (1986)

One in five Australians aged over 16 has a mental health disorder (1). Approximately one in twenty-two (or just over one million people) has an eating disorder (2, 3). Despite significant structural redress since the late twentieth century of the human rights violations long experienced by individuals with mental illness—from deinstitutionalisation to national civil rights enquiries and in more recent decades, advocacy initiatives designed to increase visibility and prompt clinical service reform (4–6), there is significant work still to be done (7). Actual and perceived stigma, as well as actual and anticipated discrimination causes immense personal suffering and continues to impede help-seeking, contributing to poorer prognosis and increased burden on the individual, their carers and the healthcare system (8–15). The 2022 National Survey of Mental Health-Related Stigma and Discrimination, conducted by the Behavioural Economics Team of the Australian Government (BETA) in partnership with the National Mental Health Commission, aims to inform the Australian National Stigma and Discrimination Reduction Strategy and is the first of four such surveys conducted since 1995 to include eating disorders (16, 17). This commentary provides a brief outline of the findings.

Australian residents (n = 7873) aged 18 and over (51% female, 49% male) were recruited via market research databases or random digit dialling to complete the survey online or over the phone. This involved reading and responding to two short vignettes (see Additional File 1) describing a person experiencing symptoms of a mental health disorder. Despite over half the respondents (53%) having experienced their own lifetime mental health disorder (17) (typically depression or anxiety), results show mental illness stigma and discrimination remain considerable in the community.

In interpreting these results, it is important to make the distinction between stigma and discrimination. The word ‘stigma’ comes from the Latin *‘stigmat’*, translated roughly to ‘mark’ or ‘stain’, and refers to negative beliefs about a person or group of people who have done something or display some characteristic society does not approve of (18, 19). ‘Discrimination’ is the prejudicial treatment of or the act of making an unjustified distinction between people based on a group or category to which they belong (e.g., race, gender or disability) (18, 19).

Eating disorders have been shown to be among the most stigmatised of the mental disorders (20, 21), largely due to volitional attribution. That is, the perception that eating disorders are personal ‘choices’, and thus in some way desirable or pleasurable, rather than life-threatening illnesses marked by anxiety, shame, isolation, hopelessness, developmental derailment and high rates of suicidal thinking (20–27). A multitude of factors contribute to this stereotype, including reductionist media representations of the illness (for example, a review of 252 articles published in seven US newspapers found 48% of articles about eating disorders ran in arts and entertainment sections, the majority of which featured young, white females and mentioned mainly environmental causal factors. Only 8% were presented in a medical context (28)). Studies based on attribution theory, which suggest enhanced understanding of the root cause of an illness will lead to increased acceptance (29), show that shifting of community perceptions of mental illness from the individual to the biogenetic in fact does little to improve the status quo and can actually exacerbate fear of mental illness and its perceived intractability (30, 31). Therefore, the perceived personal attribution associated with eating disorders and the lack of seriousness with which they are viewed (20, 21, 32) may mean that compared to other mental illnesses people with eating disorders face less discrimination (i.e., their illness is less feared) in the community, despite higher rates of stigma (they are considered more responsible for their behaviour).

## Findings

Consistent with previous research (20, 21), respondents in the national survey were overall less likely to *discriminate against* people with eating disorders than other mental health disorders, but on several measures more likely to *stigmatise* people with eating disorders. The latter included agreeing or strongly agreeing that ‘it is their own fault people with [eating disorders] are in this condition’ (11%) (highest of the disorders surveyed), that ‘[eating disorders] are not a real medical illness’ (11%) (compared to 7% for depression and 6% long-term schizophrenia), ‘[eating disorders are] a sign of personal weakness’ (12%) and ‘people with [eating disorders] could snap out of it if they wanted’ (16%) (highest of the disorders surveyed). Just over half of respondents (56%) said they would feel pity for an individual with an eating disorder (17) (see Additional File 2. Proportion of respondents agreeing with each public stigma statement). Contrarily, respondents were much less likely to feel scared of people with an eating disorder than they were people with other mental disorders (8% compared with 40% for Borderline Personality Disorder and 36% for long-term schizophrenia), and thought people with eating disorders were the least unpredictable of all individuals with mental disorders (15%).

On discrimination measures, 58% of respondents would ‘probably or definitely not’ be willing to have someone with an eating disorder look after their children (77% would ‘probably or definitely not’ be willing to have someone with depression look after their children and 94% someone with long-term schizophrenia), 42% would ‘probably or definitely not’ be willing for someone with an eating disorder to marry into their family (compared with 63% for someone with bipolar disorder and 84% for someone with long-term schizophrenia), 25% would ‘probably or definitely not’ be willing to work closely with someone with an eating disorder and 23% would ‘probably or definitely not’ be willing to make friends with someone with an eating disorder (62% of respondents would ‘probably or definitely not’ be willing to make friends with someone with long-term schizophrenia) (see Additional File 3 Proportion of respondents unwilling to engage in the activity with the person described in the vignette). People were more likely to agree that individuals with eating disorders should receive treatment against their will (23%) than should individuals with bipolar disorder (21%) or depression (19%).

The survey also asked those respondents with recent (past 12 months) personal lived experience about their own unfair treatment or stigmatisation. Across all diagnostic groups the rates of personal experience of unfair treatment were extremely high, including eating disorders. 77% of those with lived experience of an eating disorder reported experiencing unfair treatment either in private or work life (e.g., friends and family avoiding, judging or being dismissive of the person; being denied opportunities at work) or by healthcare professionals (e.g., healthcare professional was dismissive, judgemental, or prescribed medication ‘without adequate explanation, information, consultation or attempt to discuss alternatives’). Of people with their own 12-month experience of a mental health disorder, 42% reported avoiding seeking healthcare due to anticipated stigma and 78% reported concealing or hiding their mental health problem from others due to anticipated stigma (17).

It is striking to compare the results from this national survey to those reported by its predecessor, the 2011 *National Survey of Mental Health Literacy and Stigma* (16), particularly in light of previous research showing increased discrimination associated with a shift to biogenetic or external framing. In 2011, 19.7% of respondents agreed or strongly agreed that individuals with depression could snap out of it they wanted. By 2022, the number who viewed depression as under personal control had halved. However, discrimination increased fivefold; five times as many respondents were unwilling to make friends with individuals with depression (25% in 2022 vs 5% in 2011) and twice as many were unwilling to have someone with depression marry into their family (52% in 2022 vs 25.6% in 2011).

Similarly, in 2011, 11.7% of respondents agreed or strongly agreed that individuals with long-term schizophrenia could “snap out of it” if they wanted compared with 10% in 2022. However, in 2022, respondents were much less willing to make friends with an individual with schizophrenia (62% vs 19.8% in 2011) or have someone with schizophrenia marry into their family (84% vs 44% in 2011). As this was the first time eating disorders were surveyed, it is not possible to comment on how public perceptions of the illness may have changed over time. Interestingly, across all diagnostic groups the younger age bracket of 18–44 was the most likely to endorse stigmatising beliefs about people with a mental illness (17).

## Discussion

It is clear from these results that significant prejudice still exists when it comes to mental health disorders (even on the part of individuals with their own lived experience), and eating disorders are no exception. There has been an alarming increase in reported discrimination against people with mental illness since the last survey a decade ago (eating disorders were not included in the 2011 survey so it is unclear whether the high rates reported represent an increase). Survey results indicate that shifting perceptions around the personal control one is perceived to have over one’s illness (such as has occurred in Schizophrenia and Depression) is insufficient to reduce discriminatory behaviour against people with a mental illness.

An important limitation of the National Survey methodology is the use of short lay vignettes which cannot adequately capture detailed symptoms and impacts of a disorder and is particularly problematic for eating disorders. Here, the case vignette presents one type of presentation (a restrictive eating disorder rather than more common presentations of Bulimia Nervosa or Binge Eating Disorder) and describes behavioural symptoms relating to food and exercise only (see Additional File 1. Vignettes). This stands in marked contrast to the vignettes presented for the other mental disorders. It is the only vignette not to contain any mention of distress and/or emotional state, which may have minimised the perceived burden on the individual, contributing to the perception that the illness is self-imposed. Lack of conveyed complexity reflects a global misunderstanding of eating disorders and confounds numerous national and global health surveys, including, vitally, both the Global and Australian Burden of Disease studies, which use rudimentary behavioural descriptions of AN and BN rated by the general public to assign disability weighting and subsequent DALY estimates (33).

As a lived experience researcher and two clinician researchers with a combined 80 years’ experience of eating disorders, it is confronting to comprehend the degree to which our community still misunderstands and makes assumptions about the competency or likability of people with mental illness, an illness which after all exists on a spectrum that most will visit at some point in their lifetime (17, 34). But we hold this information alongside considerable hope. Large majorities of respondents in the 2022 survey agreed that it is just as important to have access to affordable mental healthcare as physical healthcare in Australia (91%), that more needs to be done to eliminate discrimination towards people affected by mental health problems (83%), and that people who intentionally harm themselves are just as deserving of medical treatment as those who have an accident (84%). This is a significant shift from decades past.

## Conclusions

In 2022, individuals with eating disorders still shoulder significant stigma. Mental illness is a painful cross to bear without the added cruelty of the loneliness and isolation stigma and discrimination engenders. As lived experience voices are increasingly amplified and greater investment made into eating disorder treatment and research, it will be integral to consider the complex communication and ethical challenges of stigma and discrimination reduction strategies. The promotion of compassion within nuanced psychoeducational frameworks will help ensure fruitful public discourse around eating disorders and a healing which must occur at the societal level.

## Supplementary Information

**Additional file 1.** Vignettes.

**Additional file 2.** Proportion of respondents agreeing with each public stigma statement.

**Additional file 3.** Proportion of respondents unwilling to engage in the activity with the person described in the vignette.

## Data Availability

All data reported in this study is drawn from the National Mental Health Commission’s report, the National Survey of Mental-Health Related Stigma and Discrimination (17).
