# Supplementary material for: Public perceptions of people with eating disorders: Commentary on results from the 2022 Australian national survey of mental health-related stigma and discrimination
Source: J Eat Disord. 2023 Apr 16;11:62. doi: 10.1186/s40337-023-00786-z (PMC10108519; doi:10.1186/s40337-023-00786-z)
Supplement: Supplementary file 1 — Additional file 1. Vignettes. [file 40337_2023_786_MOESM1_ESM.docx]

**Supplementary File 1.** Vignettes

The person is 18 years old. They have been experiencing highly distressing emotions. In response, they have been repeatedly harming themselves, leaving scars. They don’t know how else to cope with their distress but also don’t want to die. They have been covering their arms to hide the scars, even on hot days.

The person is 24 years old. They sometimes have angry outbursts at their workplace and recently lost their job because of these problems. They also have angry outbursts with friends and family. They enter new short-term relationships quickly, again and again. They are terrified each new partner will leave them, which makes them lash out at their partners when they feel unloved. Their relationships often seem to end with a fight or with arguments. They tend to find people either exceptionally admirable or terrible. Their mood is unstable and they feel like nobody understands them. They often feel like they are empty inside.

The person is 24 years old. They go to the gym twice a week to keep their fitness levels up. A few months back they started to feel that they looked heavier than other people at the gym. They also noticed a lot of posts on Facebook about weight loss. They decided to follow a low calorie diet – at first limiting their food intake for a couple of months, but then dropped to very low calories. They weigh themselves twice a day and spend most of their time thinking about food and weight. They have come to the attention of their boss, who is concerned that they have been having trouble concentrating at work.

The person is 24 years old. In the past there were times when they felt very sad and low without there being a specific reason for it. In contrast to this and to their usual behaviour, they are currently in an exceptionally good mood without any specific reason. They act very impulsively and erratically. They speak rapidly and tell others that they are having lots of new ideas and thoughts. They will often wake up earlier than usual but still feel bursting with energy. They sometimes manage without any sleep and still don’t feel tired. Unusually for them, they have been spending a lot of money recently, including buying a new car. Their family is concerned that they are spending more money than they can afford.

The person is 24 years old. They would really like to make more friends but they are scared that they will do or say something embarrassing when they are around others. They rarely say a word in work meetings and they become incredibly nervous, tremble, blush and feel like they might vomit if they have to answer a question or speak in front of their workmates. They are quite talkative with their close relatives, but they become quiet if anyone they don’t know well is present. They know their fears are unreasonable but they can’t seem to control them and this really upsets them.

The person is 38 years old. They are always on their own and are often seen sitting in the park talking to themselves. At times, they stand and move their hands as if to communicate to someone in nearby trees. They rarely drink alcohol. They speak using uncommon and sometimes made-up words. At times, they accuse shopkeepers of giving information about them to other people. Their landlord complains that the person will not let them clean the room, which is increasingly filled with glass objects. The person says they are using these "to receive messages from space". They have not worked a paid job for years.

The person is 24 years old. They have been feeling unusually sad and miserable for the last few weeks. Even though they are tired all the time, they wake up early and can’t get back to sleep. They don’t feel like eating and have lost weight. They can't keep their mind on their work and put off making decisions. They used to love playing guitar, but now they aren’t interested in it. Even day-to-day tasks seem too much for them. This has come to the attention of their boss, who is concerned about this person’s lowered productivity.

The person is 24 years old. Since finishing school, they have had a few temporary jobs but they are currently unemployed. Over the last six months, they have stopped seeing friends and they have begun locking themselves in their bedroom. They often walk about their bedroom through the night. When alone, they sometimes shout and argue as if someone else is there. They are afraid to leave home because they think the neighbour is spying on them. They do not take recreational drugs.”
