# Supplementary material for: Public perceptions of people with eating disorders: Commentary on results from the 2022 Australian national survey of mental health-related stigma and discrimination
Source: J Eat Disord. 2023 Apr 16;11:62. doi: 10.1186/s40337-023-00786-z (PMC10108519; doi:10.1186/s40337-023-00786-z)
Supplement: Supplementary file 2 — Additional file 2. Proportion of respondents agreeing with each public stigma statement. [file 40337_2023_786_MOESM2_ESM.pdf]

Additional File 1. Proportion of respondents agreeing with each public stigma statement (2022 National MentalHealth-Related Stigma and Discrimination Survey)

Statement	Long-term Schizophrenia	Borderline Personality Disorder	Early Schizophrenia	Bipolar Disorder	Non-suicidal Self-injury	Depression	Eating Disorder	Social Phobia
It is their own fault that people with this problem are in this condition	4%	8%	5%	8%	6%	6%	**11%**	6%
This problem is not a real medical illness	6%	10%	7%	10%	7%	7%	**11%**	14%
People with this problem will never recover enough to have good quality of life	14%	10%	9%	7%	7%	5%	**5%**	7%
This problem is a sign of personal weakness	9%	14%	10%	9%	14%	9%	**12%**	13%
People with this problem could snap out of it if they wanted	10%	16%	10%	10%	11%	10%	**16%**	11%
People with this problem are dangerous	31%	44%	27%	19%	20%	8%	**6%**	6%
I feel scared of people with this problem	36%	40%	29%	18%	20%	7%	**8%**	6%
People with this problem should be forced into treatment with their doctor even if they don't want to	36%	30%	32%	21%	34%	19%	**23%**	11%
People with this problem are unpredictable	68%	74%	62%	67%	44%	29%	**15%**	16%
I feel pity for people with this problem	68%	57%	63%	49%	63%	59%	**56%**	52%

Note. Each proportion includes people who responded either 'strongly agree' or 'agree' to the statement in relation the mental health problem listed in the column.
Source: National Mental Health Commission (17). Reprinted with permission from the Behavioural Economics Team of the Australian Government.
