# Supplementary material for: Public perceptions of people with eating disorders: Commentary on results from the 2022 Australian national survey of mental health-related stigma and discrimination
Source: J Eat Disord. 2023 Apr 16;11:62. doi: 10.1186/s40337-023-00786-z (PMC10108519; doi:10.1186/s40337-023-00786-z)
Supplement: Supplementary file 3 — Additional file 3. Proportion of respondents unwilling to engage in the activity with the person described in the vignette. [file 40337_2023_786_MOESM3_ESM.pdf]

Additional File 2. Proportion of respondents unwilling to engage in the activity with the person described in the vignette (2022 National Mental Health-Related Stigma and Discrimination Survey)

Activity	Long-term Schizophrenia	Borderline Personality Disorder	Early Schizophrenia	Bipolar Disorder	Non-suicidal Self-injury	Depression	Eating Disorder	Social Phobia
Move next door to someone with this problem	64%	62%	55%	41%	39%	34%	**26%**	20%
Spend an evening socialising with someone with this problem	63%	55%	43%	37%	29%	25%	**22%**	15%
Make friends with someone with this problem	62%	59%	45%	40%	32%	25%	**23%**	14%
Work closely with someone with this problem	66%	61%	48%	47%	35%	29%	**25%**	18%
Have someone with this problem marry in to family	84%	82%	71%	63%	61%	52%	**42%**	29%
Have them look after your children	94%	91%	90%	81%	81%	77%	**58%**	40%

Note. Each proportion includes people who reported being 'definitely not' or 'probably not' willing. Item 'Have them look after your children' excludes people who responded 'Not applicable'.
Source: National Mental Health Commission (17). Reprinted with permission from the Behavioural Economics Team of the Australian Government.
